# Supplementary material for: A Multimodal Platform for Simultaneous T-cell Imaging, Defined Activation, and Mechanobiological Characterization
Source: Cells. 2021 Jan 25;10(2):235. doi: 10.3390/cells10020235 (PMC7910839; doi:10.3390/cells10020235)
Supplement: Supplementary file 1 [file cells-10-00235-s001.zip › Supplementary Materials/Supplementary Materials.pdf]

Supplementary Materials

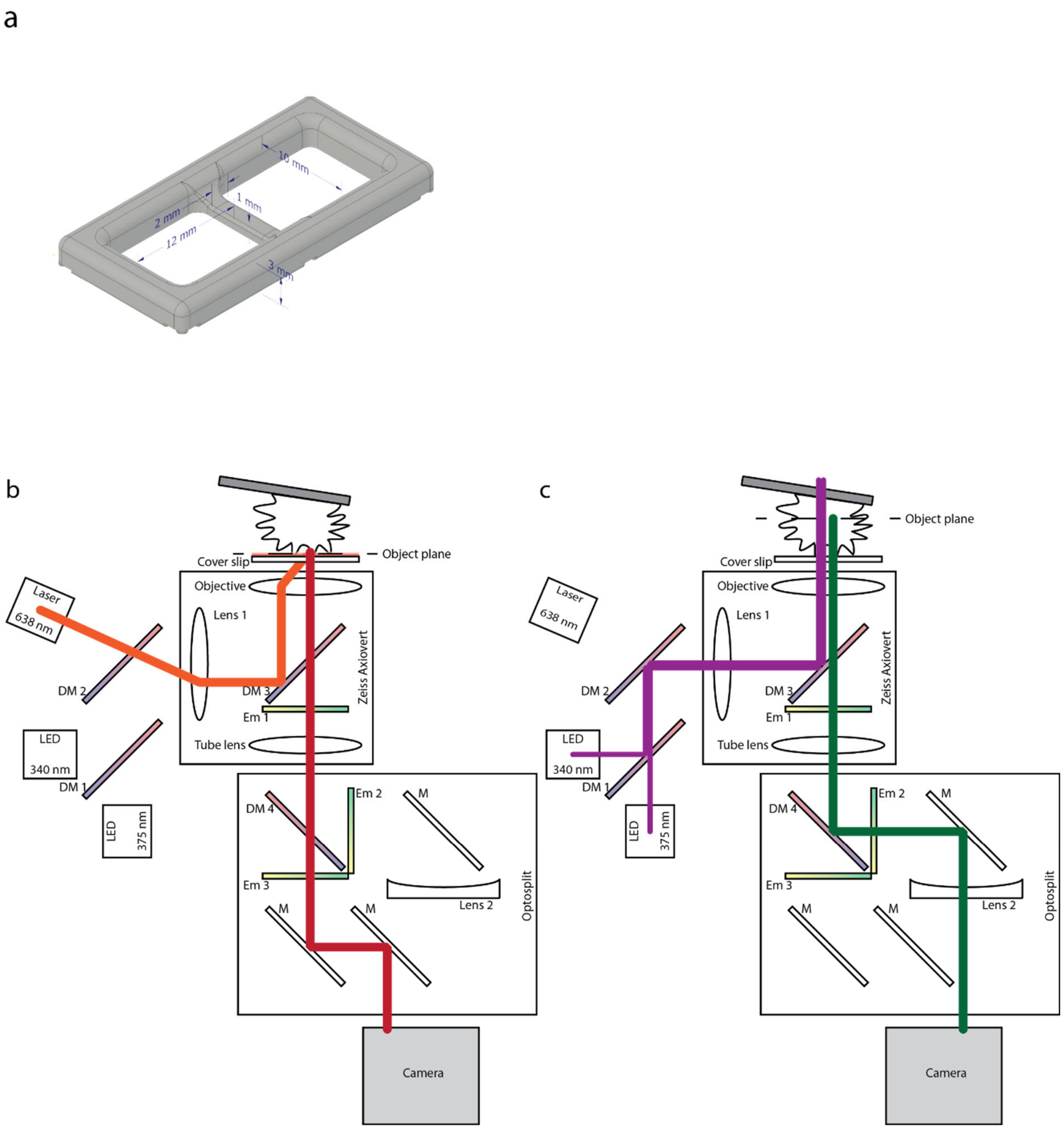

Supplementary Figure 1: **Sketches of the setup and the sample chamber.** a) drawing of the frame which separates the storage and the measurement chamber. b) and c) show the beam paths for TIRF imaging and for Fura-2 imaging, respectively. DM: dicroic mirror; EM: emission filter; M: mirror. Lens 1 denotes the lens system of the epi-illumination unit of the Zeiss micoscope. Lens 2 was a plan-concave lens with f=-100mm to shift the focal plane for calcium imaging. For TIRF illumination, we tilted the 640nm laser beam with respect to the optical axis, thereby shifting the beam parallel to the optical axis in the back focus of the objective.

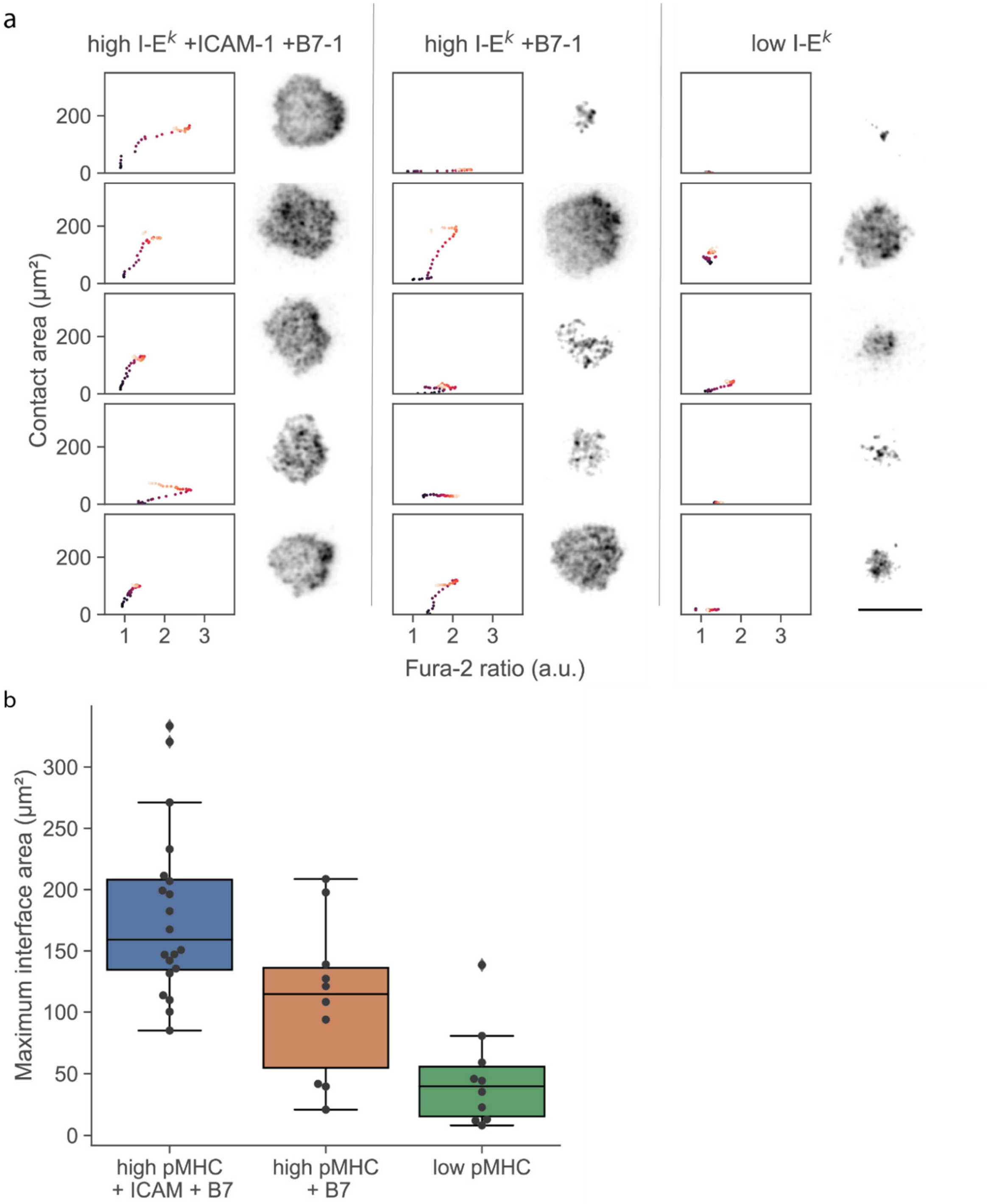

Supplementary Figure 2: **Examplary images and quantiative analysis of T cells contacting SLBs of indicated functionalization.** a) The parametric plots show the single cell contact area versus the Fura-2 signal. Colors indicate the time from t = -48 s (dark violet) to t = 100 s (bright yellow) relative to the calcium flux. Corresponding images of the TCR channel in TIRF configuration at t = 100s. Scalebar, 10μm. b) Maximum contact area for T cells delivered to SLBs functionalized with high I-E^k/MCC + ICAM-1 + B7-1 (blue, n=20 cells), high I-E^k/MCC + B7-1 (orange, n=10 cells), and low I-E^k/MCC (green, n=10 cells). Data are shown as Whisker box plots indicating the interquartile range (box), median (line), and the individual data points corresponding to single cell (circles).

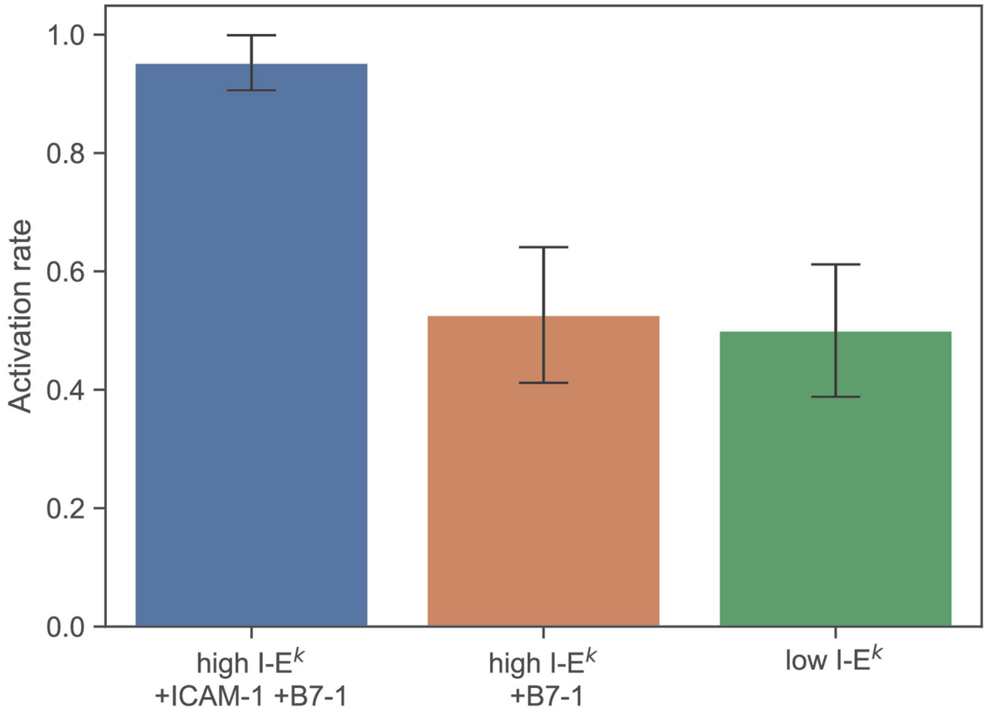

Supplementary Figure 3: **Activation rate for T cells delivered via the AFM cantilever to SLBs.** We quantified the percentage of T cells activated during the time course of the experiment for SLBs functionalized with the three indicated proteins. Error bars indicate standard error of the mean. Data are shown for n=21, n=19, n=20 cells (left to right).

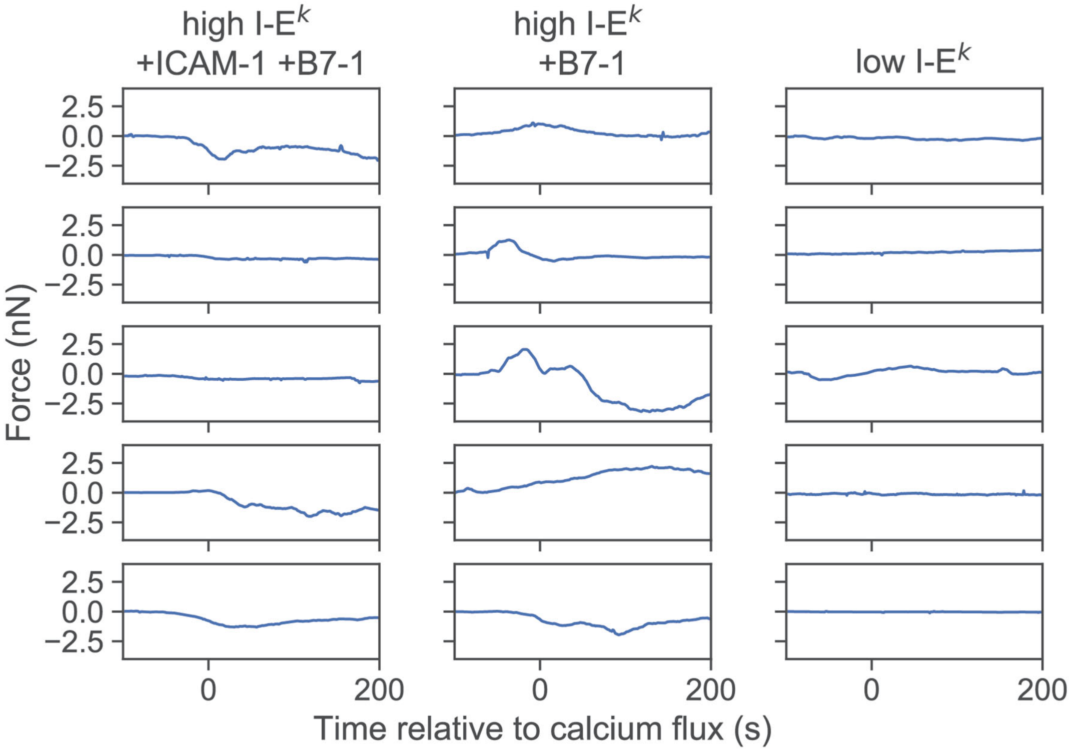

Supplementary Figure 4: **Deflections of the AFM cantilever due to pushing and pulling of single T cells.** Representative deflection traces from individual cells. All traces were synchronized with respect to the calcium-flux. Colors indicate different experiments.

Supplementary CAD files: **CAD drawings of the frame:** The stl-files provide CAD drawings of the top and bottom parts of the mold to produce the frame of the sample chamber.

Supplementary Video 1: **Exemplary data showing the approach of one T cell to an activating SLB functionalized with I-E^k/MCC, ICAM-1 and B7-1**. The video shows simultaneous recordings of the TCR channel (top left), the Fura-2 ratio (top right) and the force applied by the T cell on the AFM cantilever (bottom). Time-point zero is defined via the half-maximum value of the calcium signal. Scale bar, 10µm. Video uses the H.265 codec.
